# Impact of chronic comorbidities on psychological and social status in COVID-19 patients

**DOI:** 10.3389/fpsyg.2026.1820126

**Published:** 2026-06-15

**Authors:** Ping Wu, Zixin Jian, Shuxiang Yang, Haiyu Liu, Qiong Luo, Qianyuan Zhang, Qixian Zheng, Suyun Zhang, Qiong Lin, Xiangqi Chen

**Affiliations:** 1 Department of Respiratory and Critical Medicine, Fujian Medical University Union Hospital, Fuzhou, China; 2 Fujian Medical University, Fuzhou, China; 3 Department of Oncology Medicine, Fujian Medical University Union Hospital, Fuzhou, China; 4 Department of General Medicine, Fujian Medical University Union Hospital, Fuzhou, China; 5 Department of Internal Medicine, Fujian Medical University Union Hospital, Fuzhou, China

**Keywords:** chronic diseases, COVID-19, Medical Coping Modes Questionnaire (MCMQ), psychological and social status, Social Support Rating Scale (SSRS)

## Abstract

**Objective:**

To investigate the psychological and social status in patients with COVID-19 accompanied by common chronic diseases compared to patients without these chronic conditions. Additionally, the study aims to explore the relevant influencing factors, providing objective data and scientific theoretical basis for the psychological status of COVID-19 patients with common chronic diseases and improvement of their quality of life.

**Methods:**

Sixty patients diagnosed with COVID-19 and admitted to the Department of Respiratory and Critical Care Medicine in Fujian Medical University Union Hospital, between January 1, 2023, and March 31, 2023, were enrolled. The Hospital Anxiety and Depression Scale (HADS), Medical Coping Modes Questionnaire (MCMQ), Social Support Rating Scale (SSRS), and Type D Personality Scale (Ds-14) were used for the investigation. Admission complete blood count, blood biochemistry, and C-reactive protein (CRP) data were collected to calculate the neutrophil-to-lymphocyte ratio (NLR), platelet-to-lymphocyte ratio (PLR), and C-reactive protein-to-albumin ratio (CAR). The prevalence of anxiety and depression was compared between patients with COVID-19 accompanied by chronic diseases and those without, and coping styles, social support, Type D personality, and CAR were analyzed and compared between the two groups.

**Results:**

Among 60 COVID-19 patients, 46 had accompanying chronic diseases, 14 patients had no accompanying chronic diseases. Patients with chronic diseases had higher anxiety and depression scores than those without. Moreover, there were statistically significant differences between the two groups in MCMQ, SSRS, and CAR (*p* < 0.05). And we found that, among patients with COVID-19, Type D personality was associated with the co-occurrence of anxiety and depression, as well as with higher anxiety scores. and elevated CAR levels was associated with the co-occurrence of anxiety and depression.

**Conclusion:**

The presence of common chronic diseases significantly influenced the coping styles and social support profiles of COVID-19 patients. Different psychosocial factors were associated with the occurrence and severity scores of anxiety and depression in patients.

## 1 Introduction

Since its emergence in 2019, the novel coronavirus disease 2019 (COVID-19) has rapidly spread across the globe, posing a significant threat to human health and safety. This situation warrants ongoing attention and the implementation of sustained preventive measures. To mitigate the transmission of the virus, many nations have instituted various public health interventions, including quarantine, cessation of work, and the cancelation of large gatherings. These measures have profoundly impacted daily life and work routines, leading to an array of psychological issues associated with COVID-19, such as anxiety, depression, stress, and insomnia. These adverse psychological states can detrimentally affect treatment outcomes and may be linked to poorer prognoses (Alshammari and Alshammari, 2021; Tsai et al., 2021). Consequently, the psychological impact of COVID-19 on individuals should not be overlooked.

Patients with chronic diseases represent a particularly vulnerable population, and psychological symptoms are more prevalent among them (Liu et al., 2021). Furthermore, they are at increased risk of contracting COVID-19 (CDC COVID-19 Response Team, 2020). Research indicates that individuals with chronic conditions exhibit a higher prevalence of anxiety and depression compared to the general population (Chen et al., 2017). Additionally, COVID-19 can lead to systemic hyperinflammation, which is a possible contributing factor to the development of various chronic neuropsychiatric complications in COVID-19 survivors (Costanza et al., 2022). The COVID-19 virus may gain access through angiotensin-converting enzyme 2 receptors on endothelial cells of cerebral vessels, and also likely by crossing the damaged blood–brain barrier, which is highly susceptible to peripheral immune changes and to the characteristic cytokine storm of COVID-19 (Tang et al., 2021). Moreover, SARS-CoV-2 may directly invade and instigate inflammatory processes within the nervous system, exacerbating psychosomatic symptoms in affected individuals (Grolli et al., 2021). Beyond these physiological mechanisms, while social isolation is widely employed as an effective strategy for preventing infection, older adults living in isolation may experience further deterioration of their mental health due to both social seclusion and pre-existing health conditions (McElroy-Heltzel et al., 2022).

The mental health of these patients is shaped by a complex mix of psychosocial and biological factors. However, how these factors interact in the context of COVID-19 remains not fully understood. Previous studies have suggested that female sex is associated with increased risks of anxiety, depression and insomnia during the COVID-19 pandemic (Yuan et al., 2022). Older patients may experience greater psychological distress, which And previous studies have shown that certain personality and coping styles are closely linked to psychological distress (Eisenbeck et al., 2021; Stals et al., 2024; Grinberg and Sela, 2026). For example, a study found a strong association between Type D personality and higher levels of depression, anxiety, and stress (Grinberg and Sela, 2026). Similarly, an individual’s coping style largely determines how they manage illness-related stress. Research shows that avoidant coping is strongly tied to greater anxiety and depressive symptoms, while a meaning-centered coping approach strongly predicts lower levels of stress, anxiety, and depression (Eisenbeck et al., 2021; Stals et al., 2024). Social support also acts as an important buffer. During the pandemic, strong perceived social support has been inversely associated with mental health problems, and those who report higher support generally experience less anxiety, depression, isolation and insomnia (Nisar et al., 2021; Zhao et al., 2022). Beyond these psychosocial factors, biological markers of systemic inflammation also play a significant role. The C-reactive protein to albumin ratio (CAR) serves as a combined inflammatory marker that reflects both CRP and albumin levels (Liang et al., 2017). Changes in these acute-phase proteins are closely linked to disease severity, and in certain clinical scenarios CAR has shown better prognostic value than traditional inflammatory markers (Póvoa et al., 2020; Güney et al., 2021; Kalenderoglu et al., 2024). Recent evidence also indicates a strong positive correlation between elevated CAR levels and depressive symptoms (Lin et al., 2025). To date, there is a lack of literature addressing the psychological and social status, as well as their prevention and management, among individuals with and without chronic diseases associated with COVID-19. Quantifying such psychosocial and biological determinants is important, as it enables doctors to move beyond mere symptom control and target the core drivers of psychological problems. To address this gap, this study aimed to investigate the differences in psychological and social status between COVID-19 patients with and without chronic diseases in Fujian, China. Furthermore, it will assess and compare key associated factors, such as coping styles, social support scores, Type D personality, and C-reactive protein to albumin ratio with anxiety and depression. Using regression analyses, the study ultimately seeks to uncover the associations of these variables with both the onset and the severity of anxiety and depression. This research seeks to identify independent risk factors influencing the mental health of these patients, thereby providing a scientific foundation for the early psychological support and clinical management of individuals with chronic diseases who are at elevated risk for infection with SARS-CoV-2.

## 2 Materials and methods

### 2.1 Participants

This study included a cohort of sixty patients diagnosed with COVID-19, Inclusion criteria: (1) Laboratory-confirmed COVID-19 diagnosis, verified by positive SARS-CoV-2 nucleic acid detection via RT-PCR testing of respiratory specimens. (2) Absence of other acute respiratory infections (e.g., influenza A or B). Exclusion criteria: (1) Incomplete clinical or laboratory data. (2) Severe cognitive impairment or inability to complete questionnaires. (3) Refusal or inability to provide written informed consent. These patients were admitted to the Department of Respiratory and Critical Care Medicine at the Union Hospital of Fujian Medical University between January 1 and March 31, 2023. Clinical data were collected for each patient, including sex, age, medication use, and the type and number of chronic diseases. The chronic disease group included patients with one or more of the following conditions: hypertension, diabetes, chronic respiratory diseases (e.g., chronic obstructive pulmonary disease, Bronchiectasis), cardiovascular diseases (e.g., Atherosclerotic heart disease) and cerebrovascular diseases (e.g., cerebral infarction).

### 2.2 Ethics statement

The studies involving humans were approved by the Union Hospital of Fujian Medical University Ethics Committee (ID: 2023KY228). The participants provided their written informed consent to participate in this study.

### 2.3 Investigation process

**Figure 1:**
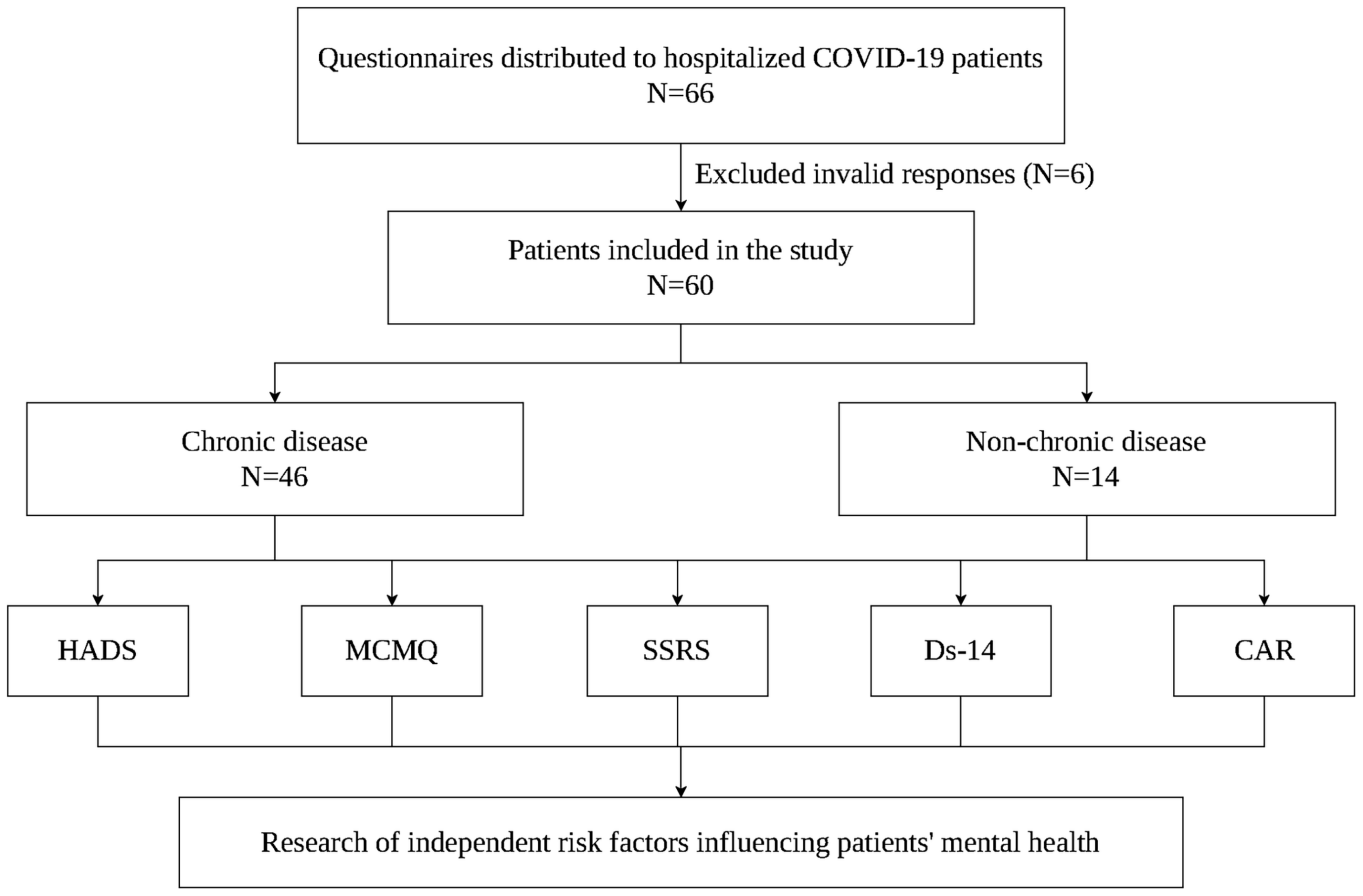
Flowchart of this study.

All questionnaires were administered within 24 h after COVID-19 diagnosis or hospital admission, prior to the initiation of specific treatment interventions, to ensure consistency in assessment timing across participants. A structured, one-on-one questionnaire was administered to the hospitalized COVID-19 patients by trained medical personnel. Before the survey initiation, informed consent was obtained from each participant, and standardized instructions on completing the questionnaire were provided. For patients unable to complete the questionnaire independently, the researcher read the questions aloud and recorded the responses on their behalf. Those who could manage the self-administered form did so independently; however, assistance was provided to individuals experiencing difficulties, ensuring that clarifications were offered for any ambiguous items and consistency in the content of the explanations. Upon collection, the investigators thoroughly reviewed the questionnaires to confirm their completeness. Any incomplete questionnaires were returned to the patients for finalization. In total, 66 questionnaires were distributed, yielding 60 and 6 invalid responses, resulting in a valid recovery rate of 90.91%. The primary reason for invalid responses was non-cooperation from patients, with some expressing that the number of questions was excessive and challenging, leading to withdrawal during the process. All study questionnaires were completed during the study period (January 1 to March 31, 2023). Additionally, the initial routine blood tests conducted on the COVID-19 patients upon admission were recorded, and the C-reactive protein to albumin ratio (CAR) was subsequently calculated. A comprehensive flowchart of this study is presented in Figure 1.

### 2.4 Investigation tools

#### 2.4.1 Hospital Anxiety and Depression Scale (HADS)

Hospital Anxiety and Depression Scale (HADS) (Lam et al., 1995): The HADS comprises 14 items, divided into two subscales of 7 items each, specifically designed to assess anxiety and depression. A score of 11 serves as the threshold for identifying significant anxiety and depressive symptoms. Previous studies have confirmed the validity of the Chinese version of the HADS. In our study, anxiety/depression score higher than 8 is considered indicative of anxiety or depression.

#### 2.4.2 Medical Coping Modes Questionnaire (MCMQ)

Medical Coping Modes Questionnaire (MCMQ) (Feifel, 1987): Developed by Feifel in 1987 and translated into Chinese by Jiang Qianjin et al., the MCMQ contains a total of 20 items organized into three dimensions representing distinct cognitive-behavioral coping styles: confrontation, avoidance, and submission. Responses are rated on a 4-point Likert scale, and the questionnaire demonstrates strong reliability and validity.

#### 2.4.3 Social Support Rating Scale (SSRS)

Social Support Rating Scale (SSRS) (Cauce et al., 1982; Zhao et al., 2020): Established by Xiao Shuiyuan in 1986, the SSRS has been validated for reliability and effectiveness in measuring social support. It includes ten items scored on a 4-point Likert scale across three dimensions: objective support, subjective support, and the utilization of social support. Higher scores indicate greater perceived support.

#### 2.4.4 Type D Personality Scale (Ds-14)

Type D Personality Scale (Ds-14) (Mols et al., 2012): The Ds-14 scale, developed by Denollet, has been validated in its Chinese version for reliability and effectiveness as a psychological assessment tool. It consists of two subscales: negative affect (NA) and social inhibition (SD), each containing seven items. Responses are scored on a five-point scale ranging from “0 = not at all true” to “4 = very much true.” Participants with NA ≥ 10 and SD ≥ 10 are classified as having a Type D personality.

The Hospital Anxiety and Depression Scale (HADS), Medical Coping Modes Questionnaire (MCMQ), and Type D Personality Scale (Ds-14) used in this study have been previously translated into Chinese and validated in Chinese populations, demonstrating acceptable reliability and validity (Yu et al., 2008; Yang et al., 2014; Ye et al., 2017; Wang et al., 2024). The Social Support Rating Scale (SSRS) is a Chinese indigenous instrument developed by Xiao Shuiyuan and has been extensively applied in clinical and epidemiological studies in China.

### 2.5 Statistical analysis

First, clinical characteristics and psychological/social assessment scores were compared between COVID-19 patients with and without chronic diseases.

Given the relatively small sample size, a limited number of variables were included in the regression analyses. Age, sex, Type D personality, and the C-reactive protein to albumin ratio (CAR) were entered into models. The confrontation dimension of the Medical Coping Modes Questionnaire (MCMQ) was selected to represent coping style, and the total score of the Social Support Rating Scale (SSRS) was used to represent overall social support. These variables were subsequently included in both binary logistic regression analyses and multiple linear regression analyses to examine their associations with anxiety and depression outcomes. A *p*-value of <0.05 was deemed statistically significant. Data analysis was conducted using SPSS version 22.0.

## 3 Results

### 3.1 General characteristics of patients with and without chronic diseases

This study included a total of 60 patients diagnosed with COVID-19. Of these, 46 patients had pre-existing chronic diseases, while 14 did not. The baseline clinical characteristics, residential distribution (urban/rural), and medical insurance types of the two groups are presented in Table 1. Among the chronic disease group, the most prevalent conditions were hypertension (*n* = 36) and diabetes (*n* = 25). The majority of patients had either one chronic disease (*n* = 23) or two chronic diseases (*n* = 17) (Supplementary Tables S1, S2).

**Table 1:** Baseline characteristics of participants.

Character	All (*n* = 60)	Chronic disease group (*n* = 46)	Non-chronic disease group (*n* = 14)	*p*
Sex				
Male	42 (70.00%)	31 (67.40%)	11 (78.6%)	0.52
Female	18 (30.00%)	15 (32.61%)	3 (21.43%)	
Age	75.00 (69.56, 76.37)	76.50 (71.47, 78.10)	68.00 (56.88, 77.12)	0.14
Psychotropic medication use	9 (15.00%)	8 (17.39%)	1 (7.14%)	0.67
Residence(urban)	49 (81.67%)	38 (63.33%)	11 (78.6%)	0.71
Urban insurance	41 (68.33%)	33 (71.7%)	8 (57.14%)	0.48

### 3.2 Prevalence of anxiety and depression in patients with and without chronic diseases

The Hospital Anxiety and Depression Scale (HADS) was employed to assess anxiety and depression in patients with COVID-19. Patients with chronic diseases showed higher anxiety and depression scores than those without chronic diseases; however, the differences did not reach statistical significance. Anxiety symptoms were identified in 11 patients (23.9%) in the chronic disease group and 4 patients (28.6%) in the non-chronic disease group, while depressive symptoms were observed in 14 patients (30.4%) in the chronic disease group and 2 patients (14.3%) in the non-chronic disease group. Detailed results are presented in Table 2.

**Table 2:** Comparison of psychological and social status in 2 groups of patients with COVID-19.

Character	Total (*n* = 60)	Chronic disease group (*n* = 46)	Non-chronic disease group (*n* = 14)	*p*
Anxiety	15 (25.0%)	11 (23.9%)	4 (28.6%)	0.73
Depression	16 (26.7%)	14 (30.4%)	2 (14.3%)	0.31
Anxiety and depression	11 (18.3%)	9 (8.4%)	2 (14.3%)	0.96
Anxiety or depression	20 (33.3%)	16 (34.8%)	4 (4.7%)	0.91
Anxiety score	5.87 ± 3.69	5.87 ± 3.63	5.85 ± 4.04	0.99
Depression score	5.38 ± 3.67	5.61 ± 3.82	4.64 ± 3.13	0.39
Type D personality	16 (26.67%)	13 (28.26%)	3 (21.43%)	0.87
MCMQ				
Confrontation score	16.78 ± 2.37	15.96 ± 2.00	19.50 ± 1.16	<0.001
Avoidance score	14.13 ± 1.98	14.70 ± 1.86	12.29 ± 0.99	<0.001
Submission score	5.93 ± 2.56	6.63 ± 2.50	3.64 ± 0.93	<0.001
SSRS				
Objective support score	7.12 ± 1.83	7.91 ± 1.09	4.50 ± 1.22	<0.001
Subjective support score	21.38 ± 4.57	22.87 ± 4.08	16.50 ± 1.91	<0.001
Support utilization	6.67 ± 1.53	7.15 ± 1.40	5.07 ± 0.47	<0.001
SSRS Total score	35.17 ± 6.56	37.93 ± 4.45	26.07 ± 3.27	<0.001

### 3.3 Comparison of coping styles, social support, Type D personality in COVID-19 patients with and without chronic disease

The Medical Coping Mode Questionnaire (MCMQ) showed difference of the coping styles between two groups with COVID-19. The differences in confrontation, avoidance, and submission scores between the two groups were all statistically significant. Detailed results are presented in Table 2. In terms of social support, patients with chronic diseases showed significantly higher objective support scores, subjective support scores, support utilization scores, and total social support scores comparing with whom without chronic diseases. All differences between the two groups were statistically significant (*p* < 0.05). Detailed results are presented in Table 2.

In this study, the Type D Personality Scale (DS-14) was used to assess the prevalence of Type D personality among patients with COVID-19. As shown in Table 2, the proportion of patients with Type D personality was 28.26% in the chronic disease group and 21.43% in the non-chronic disease group. However, this difference was not statistically significant.

### 3.4 Relationship between CAR, NLR, and PLR in COVID-19 patients with and without chronic disease

The CAR was significantly higher in patients with chronic diseases compared with those without chronic diseases (*p* < 0.05), which have been presented in Table 3.

**Table 3:** Comparison of CAR, NLR and PLR in 2 groups of patients with COVID-19.

Character	Total (*n* = 60)	Chronic disease group (*n* = 46)	Non-chronic disease group (*n* = 14)	*p*
CAR	0.68 (0.17, 3.71)	0.76 (0.21, 1.89)	0.46 (0.09, 1.36)	0.009
NLR	4.93 (3.15, 10.53)	4.62 (2.95, 10.49)	5.61 (3.43, 10.70)	0.42
PLR	204.89 (142.56, 316.09)	193.04 (135.45, 304.37)	216.83 (163.12, 352.09)	0.72

Neutrophil-to-lymphocyte ratio (NLR): neutrophil count/lymphocyte count. Platelet-to-lymphocyte ratio (PLR): platelet count/lymphocyte count. CRP-to-albumin (CAR): CRP (mg/L)/albumin (g/L).

### 3.5 Association of coping styles, social support, Type D personality CAR with anxiety and depression

Binary logistic regression analysis was performed to explore the associations of coping styles, social support, Type D personality, and CAR with the presence of anxiety and depression. The results showed that age (OR = 1.13, *p* = 0.03) and Type D personality (OR = 9.01, *p* = 0.02) were significantly associated with the co-occurrence of anxiety and depression. We also examined the associations between anxiety or depression and these variables; however, no statistically significant relationships were observed (Table 4).

**Table 4:** Association of coping styles, social support, Type D personality and CAR with co-occurrence of anxiety and depression.

Variates	β	OR (95%CI)	*p*
Age	0.12	1.13 (1.01, 1.25)	0.03
Sex			
Male			
Female	2.16	8.64 (1.07, 69.66)	0.04
MCMQ_confrontation score	0.28	1.33 (0.71, 2.46)	0.37
SSRS total score	−0.02	0.98 (0.84, 1.15)	0.84
Type D personality			
Non–Type D			
Type D	2.20	9.01 (1.33, 61.00)	0.02
CAR	0.65	1.91 (1.01, 3.63)	0.04

Multiple linear regression analyses indicated that age (B = 0.12, *p* = 0.01) and Type D personality (B = 2.14, *p* = 0.04) were significantly associated with higher anxiety scores. In contrast, none of these variables showed significant associations with depression scores. All variables in linear regression demonstrated no evidence of multicollinearity, with variance inflation factor <3. However, no significant associations were observed between these variables and depression scores (Table 5).

**Table 5:** Association of coping styles, social support, Type D personality and CAR with score of anxiety.

Variates	B (95% CI)	*p*	VIF
Age	0.12 (0.02, 0.16)	0.01	1.11
Sex			
Male			
Female	1.75 (−0.27,3.77)	0.09	1.05
MCMQ_confrontation score	0.24 (−0.26,0.73)	0.34	1.41
SSRS total score	−0.02 (−0.18,0.15)	0.85	1.46
Type D personality			
Non–Type D			
Type D	2.14 (0.09,4.19)	0.04	1.05
CAR	0.40 (−0.29,1.10)	0.25	1.22

VIF, variance inflation factor.

## 4 Discussion

In our study, patients with COVID-19 and chronic diseases exhibited higher anxiety and depression scores compared with those without chronic diseases. Significant differences were also observed between the two groups in coping styles, social support, and the C-reactive protein-to-albumin ratio (CAR). Furthermore, among patients with COVID-19, older age, Type D personality, and higher CAR levels were associated with the co-occurrence of anxiety and depression, while Type D personality was additionally associated with increased anxiety severity scores. These findings suggest that psychological, social, and inflammatory factors may collectively contribute to mental health outcomes in patients with COVID-19, particularly among those with chronic diseases (Table 5).

COVID-19 is classified as an acute respiratory infectious disease, patients with underlying chronic conditions are particularly vulnerable due to factors such as immunocompromise and the exacerbation of pre-existing comorbidities (Chen et al., 2021). This psychological impact is often reflected in alterations to self-perception, attitudes, and overall meaning in life (Samushiya et al., 2022; Salah et al., 2023). Unfortunately, the psychological and social status in individuals with COVID-19, frequently remains overlooked. Moreover, lower muscular strength and reduced muscle mass have been associated with higher prevalence of depressive symptoms in older adults with chronic disease comorbidity (Lee and Ryan, 2020; Yang et al., 2025). Several mechanistic insights from experimental animal models of muscle atrophy further support these observations (Zhang et al., 2024). Therefore, this study aims to investigate the psychological and social status in patients with COVID-19, particularly those with chronic comorbidities in order to monitor and manage the prolonged neuropsychological and psychosocial sequelae of COVID-19.

In this study, patients with chronic diseases had higher anxiety and depression scores than those without, indicating a trend toward greater psychological distress among these individuals. Though the differences were not statistically significant. This lack of significance may be attributable to several factors. It may be attributed to the limited sample size and the small number of patients in the non-chronic disease group, which reduced the statistical power to detect differences. Despite the absence of statistical significance, the higher mean scores in the chronic disease group suggest a potential vulnerability. Patients with chronic diseases, particularly when concomitant acute illnesses are present, exhibit a heightened likelihood of experiencing anxiety and depression. It was found that older adults with comorbid chronic conditions were more prone to significant psychosomatic issues. Furthermore, the influence of physical health on mental health changes was observed to be greater than the reciprocal impact of mental health on subsequent physical health (Luo et al., 2020). Anxiety and depression are among the most prevalent psychiatric symptoms observed during the acute, prolonged, and post-acute phases of COVID-19 (Soriano et al., 2022). A study by (Xie et al., 2022), which analyzed data from 153,848 patients with COVID-19, reported an increased risk ratio of anxiety at 1.35 (95% credible interval: 1.30–1.39) and a risk ratio of depression at 1.39 (95% confidence interval: 1.34–1.43). These findings suggest that greater attention should be paid to the psychological health of patients with COVID-19, particularly those with chronic diseases.

Our research results indicated that, in the dimensions of confrontation, avoidance, and submission, the confrontation score among patients with chronic diseases was significantly lower than that of patients without chronic diseases (*p* < 0.05). And the avoidance and submission scores were markedly higher in the chronic disease group compared to their counterparts without chronic conditions (*p* < 0.05). Previous Research revealed that differing coping styles among stroke patients significantly impacted their physical functioning, social participation, and familial relationships (Li Y. et al., 2021). Similarly, elderly patients with newly diagnosed coronary artery disease and chronic comorbidities were reported to be more likely to exhibit avoidance behaviors, appetite loss, and symptoms of anxiety and depression (Zeng et al., 2021). Furthermore, a study by Qin et al. in 2023 demonstrated that a positive coping attitude significantly aided elderly patients with chronic heart failure in overcoming kinesiophobia (Qin et al., 2023). This study not only corroborates the findings of previous research but also suggests that patients tend to adopt negative or avoidant coping strategies when confronted with multiple adversities, such as COVID-19 and chronic illnesses. These findings highlight that clinicians should pay close attention to patients’ coping styles and provide appropriate psychological support for patients with COVID-19, particularly those with chronic diseases, in order to help them develop more adaptive coping strategies.

In our study, we found that individuals with chronic conditions scored significantly higher than their counterparts without chronic illnesses across several dimensions, including objective support, subjective support, support utilization, and total support scores (all *p* < 0.05). These findings may indicate that patients with chronic diseases receive greater social attention and support during the course of disease management. Social support can be categorized into three components: subjective support, objective support, and the utilization of support. Social support exerts an influence on both physical and mental health through two principal mechanisms (Cohen and Wills, 1985). The first mechanism indicates that social support generally benefits all individuals, regardless of their stress levels. The second mechanism involves stress buffering, whereby social support serves to mitigate the impacts of life stressors, particularly for those experiencing heightened stress (Gable and Bedrov, 2022). In Gil et al. demonstrated that social support is frequently correlated with enhanced adaptation to chronic illness among patients, with their perception of disease severity influenced by the adequacy of social support received (Gil et al., 1987). Subsequently, Bustamante et al. revealed that social support significantly predicted improved self-management among patients (Bustamante et al., 2018). Further research highlighted that during the COVID-19 pandemic, older adults with chronic conditions reported relatively high levels of social support (Zhang et al., 2023). McElroy-Heltzel et al. identified a relationship between perceived social support and reduced psychological distress among patients with chronic illnesses, noting that it provided a degree of resilience against the mental stress induced by pandemic-related resource constraints (McElroy-Heltzel et al., 2022). These findings suggest that higher levels of social support may help patients better cope with the dual burden of COVID-19 and chronic comorbidities. Nevertheless, despite relatively higher social support scores, patients with chronic diseases in our study still exhibited higher anxiety and depression scores, indicating that interventions targeting coping styles and social support alone may not be sufficient. Other multiple interacting biological, psychological, and social factors should also be taken into consideration, including insomnia, neurodegenerative diseases, and social isolation (Kim and Jung, 2021; Tian et al., 2023; Zhang et al., 2026).

Our study found that, the Prevalence of Type D personality was notably higher among patients with chronic diseases than among those without chronic diseases, although the difference did not reach statistical significance. Furthermore, we found that Type D personality was associated with the co-occurrence of anxiety and depression, as well as with higher anxiety severity scores in patients with COVID-19. Previous studies have shown that Type D personality is associated with both impaired physical health and declining mental health in patients with cardiovascular diseases, diabetes, and other chronic conditions (Versteeg et al., 2012; Spek et al., 2018). Further research by Shahsavarinia et al. indicated a correlation between Type D personality and infection with COVID-19, positing that stress and anxiety may serve as significant mediating factors (Shahsavarinia et al., 2021). This underscores the importance for clinicians to be vigilant regarding chronic psychological distress in these patients Some studies have demonstrated that Type D personality is associated with higher anxiety and depression scores in patients with cardiovascular diseases, fibromyalgia, and other chronic conditions (Gokcen et al., 2022; Grinberg and Sela, 2026). In addition, Type D personality has also been reported to be associated with post-stroke depression (Yin et al., 2023). These findings are consistent with the results of our study, in which Type D personality was associated with the co-occurrence of anxiety and depression, as well as with higher anxiety severity scores among patients with COVID-19. These findings suggest that Type D personality may play an important role in psychological vulnerability among patients with COVID-19, particularly in those with chronic comorbidities. And it also suggested that a potential link between Type D personality and psychological comorbidities, particularly in patients with chronic illnesses complicated by COVID-19. Given the heightened risk of negative impacts on quality of life and mental well-being among chronically ill patients with Type D personality, it is crucial to implement enhanced psychosocial interventions.

Our results revealed that patients with chronic diseases exhibited significantly higher CAR levels compared to those without chronic conditions (*p* < 0.05). Furthermore, elevated CAR levels were associated with the co-occurrence of anxiety and depression among patients with COVID-19. Previous studies have suggested that the C-reactive protein-to-albumin ratio (CAR) may serve as a useful indicator for disease assessment in chronic conditions such as COPD and chronic kidney disease (Li H. et al., 2021; Simsek et al., 2022). In addition, CAR has also been reported as an effective predictor of prognosis in patients with COVID-19 (Zavalaga-Zegarra et al., 2022). In the present study, patients with chronic diseases exhibited higher CAR levels than those without chronic diseases among patients with COVID-19. This may be attributed to the persistent low-grade systemic inflammation commonly observed in chronic diseases. Moreover, patients with chronic comorbidities are more likely to experience malnutrition and frailty, which may contribute to reduced albumin levels. Meanwhile, previous studies conducted in both Chinese and American populations have demonstrated a close association between elevated CAR levels and anxiety symptoms, which is consistent with the findings of our study (Fong et al., 2022; Jiang et al., 2025). These findings suggest that systemic inflammation may play an important role in the development of psychological distress. Persistent inflammatory activation may contribute to anxiety and depressive symptoms through mechanisms involving neuroinflammation, immune dysregulation, and alterations in neuroendocrine pathways. These findings underscore the need for clinicians to recognize the multifaceted role of CAR. Accordingly, clinicians should consider monitoring CAR and related laboratory parameters, particularly in patients with chronic comorbidities, in order to facilitate early identification of individuals at higher risk of adverse psychological outcomes and to support timely multidisciplinary interventions.

In conclusion, our study indicates that patients with COVID-19 and pre-existing chronic diseases exhibit differences in psychological and social functioning compared to those without chronic conditions. The prevalence of anxiety and depression is influenced by various factors, including age, sex, D-type personality and CAR, and other pertinent variables. These findings highlight the importance of early psychosocial interventions, tailored support strategies, and effective communication for patients with chronic conditions.

This study has several limitations. First, the sample size was relatively small and the groups were imbalanced. All patients were recruited from a single center, it may introduce potential selection bias. Second, the psychometric instruments used in this study were not specifically designed to assess psychiatric and psychological complications resulting from the interplay of chronic diseases and COVID-19. It highlighted the need for more targeted assessment tools. Third, the cross-sectional design means that all data were captured at a single time point. Therefore, baseline psychological status before COVID-19 infection was unavailable, and causal relationships could not be established. In future studies on the psychological and social status of patients with chronic diseases, larger and more balanced samples should be recruited, and potential interactions between demographic, clinical, and psychosocial factors should be explored to gain a more comprehensive understanding of their impact.

## 5 Conclusion

This study demonstrates that COVID-19 patients with chronic diseases exhibit distinct psychological and social profiles. The mental health of these patients is influenced by several factors, including coping styles, social support, CAR, and other associated variables. Consequently, implementing timely psychological and pharmacological interventions for patients with chronic diseases linked to COVID-19 may effectively mitigate the onset and progression of anxiety and depression. Such interventions could further enhance the overall survival rates and quality of life for these patients.

Nonetheless, this study is subject to certain limitations, including geographical constraints, sample size, and variations in psychometric assessment methodologies. Therefore, more comprehensive research is warranted to explore the psychosomatic characteristics of patients with novel coronavirus pneumonia who have chronic diseases. Future studies should focus on conducting robust clinical and foundational research to evaluate the effects of tailored treatment strategies on clinical outcomes and prognoses for this patient population.

## Data Availability

The raw data supporting the conclusions of this article will be made available by the authors, without undue reservation.
