# Supplementary material for: Impact of chronic comorbidities on psychological and social status in COVID-19 patients
Source: Front Psychol. 2026 Jun 15;17:1820126. doi: 10.3389/fpsyg.2026.1820126 (PMC13310906; doi:10.3389/fpsyg.2026.1820126)
Supplement: Supplementary file 1 [file Table_1.DOC]

Table S1. Distribution of chronic disease types in the chronic disease group (n=46)

| Type of chronic disease | n | % |
| --- | --- | --- |
| Hypertension | 36 | 78.3% |
| Diabetes | 25 | 54.35% |
| Respiratory diseases | 6 | 13.04% |
| Cardiovascular diseases | 6 | 13.04% |
| Cerebrovascular diseases | 3 | 6.52% |
