# Supplementary material for: Impact of chronic comorbidities on psychological and social status in COVID-19 patients
Source: Front Psychol. 2026 Jun 15;17:1820126. doi: 10.3389/fpsyg.2026.1820126 (PMC13310906; doi:10.3389/fpsyg.2026.1820126)
Supplement: Supplementary file 2 [file Table_2.DOC]

Table S2. Distribution of the number of chronic diseases among patients in the chronic disease group (n=46)

| Number of chronic diseases | n | % |
| --- | --- | --- |
| 1 | 23 | 50.00% |
| 2 | 17 | 36.96% |
| 3 | 5 | 10.87% |
| ≥4 | 1 | 2.17% |
